# Eating Disorders and Obesity: The Challenge for Our Times

**DOI:** 10.3390/nu11051055

**Published:** 2019-05-11

**Authors:** Phillipa Hay, Deborah Mitchison

**Affiliations:** 1 Translational Health Research Institute (THRI), School of Medicine, Western Sydney University, Locked Bag 1797, Penrith, NSW 2751, Australia; 2 Department of Psychology, Macquarie University, Sydney, NSW 2109, Australia; d.mitchison@westernsydney.edu.au

**Keywords:** bulimia nervosa, binge eating disorder, weight, dieting, treatment

## Abstract

Public health concerns largely have disregarded the important overlap between eating disorders and obesity. This Special Issue addresses this neglect and points to how progress can be made in preventing and treating both. Thirteen primary research papers, three reviews, and two commentaries comprise this Special Issue. Two commentaries set the scene, noting the need for an integrated approach to prevention and treatment. The empirical papers and reviews fall into four broad areas of research: first, an understanding of the neuroscience of eating behaviours and body weight; second, relationships between disordered eating and obesity risk; third, new and integrated approaches in treatment; and fourth, assessment. Collectively, the papers highlight progress in science, translational research, and future research directions.

## 1. Introduction

Public health concerns over the rising health toll resulting from weight disorders have become increasingly strident. However, as outlined in the two commentaries of this Special Issue [1,2], the concomitant mental health toll is largely ignored despite well-researched links between the physical and mental health of people living with larger bodies. Disordered eating is both an important risk and a perpetuating factor for obesity, often mediated through psychological states such as low mood or negative affect. Likewise, psychological concomitants of high Body Mass Iindex (kg/m^2^; BMI) such as body dissatisfaction and weight stigma contribute to the increasing burden of eating disorders worldwide. 

Thirteen primary research papers, three reviews, and two commentaries comprise this Special Issue. The two commentaries set the scene, calling for an integrated approach to the prevention [1] and treatment [2] of both problems. The primary papers and reviews fall into four broad areas of research: first, an understanding of the neuroscience of eating behaviours and body weight across the biopsychosocial and cultural spectrum; second, an exploration of relationships between disordered eating and obesity risk; third, new and integrated approaches in the treatment of obesity and eating disorders; and fourth, assessment in research and clinical domains.

## 2. Understanding the Neuroscience of Eating Behaviors and Body Weight 

In this Special Issue, the complexity of eating and its sociodemographic and cultural contexts is highlighted in papers ranging from investigating the impact of lifestyle and health literacy in the Roma peoples of the Czech Republic [3] to demonstrating the relationships between family functioning and obesogenic nutrient consumption [4]. In a systematic review of 20 papers [5], there was suggestive but inconclusive support for associations among some food-related parenting practices and parental high BMI.

In neurocognitive research, Edwards et al. [6] found longer electroencephalographic (EEG) measured reaction times are associated with eating disorder symptoms in individuals with a high BMI. In a preliminary study, Schmidt et al. [7] reported associations between weight status and changes in EEG patterns, which correlated with general impulsivity and food approach behaviors. Smith et al. [8] reported a differential neuronal regulation of binge type eating by a novel mechanism, neuromedin U Receptor 2 (NMUR2), which points to future treatment research. The systematic review by Imperatori et al. [9] supported a future role for neural and bio-feedback-based approaches for disordered eating behaviors, such as food craving or rumination, with a neurocognitive rationale (modulation of brain reward mechanisms) supported by empirical research. 

## 3. Exploring Relationships between Disordered Eating and Obesity Risk

Several papers investigated how disordered eating may be related to obesity risk. In addition to socio-demographic factors, Martin-Biggers et al. [10] found higher weight-related teasing, higher body dissatisfaction, and concern about a child’s weight status significantly explained maternal obesity risk, which was also associated with food insecurity and poor family food quality. The findings of Blume et al. [11] support distinct neurocognitive profiles for people with binge eating disorders (BED) in comparison with people with a high BMI without BED. In addition, they explored the impacts of food addiction symptoms which are associated with higher levels of depression in individuals with BED.

The study of special populations and people with problems across the weight spectrum can inform understanding of mechanisms of weight loss/gain and under/over-eating. Two papers in this Special Issue highlight such areas for further research. First, the Figel review [12] suggested that athletes who have suffered spinal cord injuries, who have become sedentary and are at risk of becoming overweight, may have a higher risk of poor nutrition or becoming undernourished, as seen in people with eating disorders characterized by weight loss and dietary restriction such as anorexia nervosa. Plichta et al. [13] investigated body satisfaction and nutrition in students with and without orthorexic (rigid healthy eating) tendencies. Although orthorexia may represent a new eating disorder, people with the disorder differ from people with established eating disorders in key ways, particularly in their relationship with nutrition and attitudes towards their body weight, as exemplified in this study. 

## 4. Treatments Addressing Co-Morbidity and Integrated Care

Bariatric surgery is the leading evidence-based approach in the treatment of obesity, but can it cause or exacerbate eating disorders through the inevitable state of imposed dietary restriction? In this Special Issue, Subramaniam et al. [14] reported overall improvement in mental health and eating status six months post-surgery. However, poor mental health and eating prompted by external cues prior to surgery were associated with poorer outcomes post-surgery, highlighting the need to actively address mental health and eating behavior prior to surgery. On the other hand, an effective treatment for bulimia nervosa or BED, such as cognitive behavior therapy, did little to improve metabolic physical health status in a randomized controlled trial by Mathisen et al. [15]. Nitsch et al. [16] concluded the Special Issue with a report on how to improve engagement in a new, online, integrated prevention program that addresses eating, weight, and mental health of adolescents called “Healthy Teens @ School”.

## 5. Assessment and Diagnosis

Assessment instruments and clinically relevant diagnostic schemes are important in any field. Burton et al. [17] evaluated a useful tool, the Eating Beliefs Questionnaire (EBQ), which assesses negative, positive, and permissive beliefs about eating that can contribute to eating behaviors such as binge eating. The validated EBQ can now be used in research investigations; for example, the role of potentially remedial beliefs and behaviors as indicators of obesity and eating disorder risk. Amorim Palavaras et al. [18] found that the broader definition of binge eating (with emphasis on loss of control over eating rather than quantity consumed) in the ICD-11 proved of greater utility without loss of validity in a clinical population of individuals with high BMI.

## 6. Conclusions

Collectively, these papers advance the understanding of the complex relationships between weight and eating problems, from scientific reports to translational research papers. The papers also point to the urgent need for additional research and collaboration between the two fields, which for too long have worked in parallel, rarely crossing or meeting. The papers also highlight the ways each field can learn from the other. To this end, we have initiated a collaborative University White Paper to support a new national direction, a Centre of Translational Research and Action for Eating and Weight Disorders (ASTRA—[19]). This White Paper also highlights the need for guidelines on optimal care for people with both problems. Only with such integrated endeavors can these fields jointly progress. 

## References

[1] Sonneville K., Rodgers R. (2019). Shared Concerns and Opportunity for Joint Action in Creating a Food Environment That Supports Health. Nutrients.

[2] da Luz F., Hay P., Touyz S., Sainsbury A. (2018). Obesity with comorbid eating disorders: Associated health risks and treatment approaches. Nutrients.

[3] Olišarová V., Tóthová V., Bártlová S., Dolák F., Kajanová A., Nováková D., Prokešová R., Šedová L. (2018). Cultural Features Influencing Eating, Overweight, and Obesity in the Roma People of South Bohemia. Nutrients.

[4] Jaramillo M., Burke N., Shomaker L., Brady S., Kozlosky M., Yanovski J., Tanofsky-Kraff M. (2018). Perceived Family Functioning in Relation to Energy Intake in Adolescent Girls with Loss of Control Eating. Nutrients.

[5] Patel C., Karasouli E., Shuttlewood E., Meyer C. (2018). Food Parenting Practices among Parents with Overweight and Obesity: A Systematic Review. Nutrients.

[6] Edwards C., Walk A., Thompson S., Mullen S., Holscher H., Khan N. (2018). Disordered Eating Attitudes and Behavioral and Neuroelectric Indices of Cognitive Flexibility in Individuals with Overweight and Obesity. Nutrients.

[7] Schmidt R., Sebert C., Kösling C., Grunwald M., Hilbert A., Hübner C., Schäfer L. (2018). Neuropsychological and Neurophysiological Indicators of General and Food-Specific Impulsivity in Children with Overweight and Obesity: A Pilot Study. Nutrients.

[8] Smith A.E., Kasper J.M., Anastasio N.C., Hommel J.D. (2019). Binge-Type Eating in Rats is Facilitated by Neuromedin U Receptor 2 in the Nucleus Accumbens and Ventral Tegmental Area. Nutrients.

[9] Imperatori C., Mancini M., Della Marca G., Valenti E., Farina B. (2018). Feedback-based treatments for eating disorders and related symptoms: a systematic review of the literature. Nutrients.

[10] Martin-Biggers J., Quick V., Spaccarotella K., Byrd-Bredbenner C. (2018). An Exploratory Study Examining Obesity Risk in Non-Obese Mothers of Young Children Using a Socioecological Approach. Nutrients.

[11] Blume M., Schmidt R., Hilbert A. (2018). Executive Functioning in Obesity; Food Addiction, and Binge-Eating Disorder. Nutrients.

[12] Figel K., Pritchett K., Pritchett R., Broad E. (2018). Energy and Nutrient Issues in Athletes with Spinal Cord Injury: Are They at Risk for Low Energy Availability?. Nutrients.

[13] Plichta M., Jezewska-Zychowicz M., Gębski J. (2019). Orthorexic Tendency in Polish Students: Exploring Association with Dietary Patterns, Body Satisfaction and Weight. Nutrients.

[14] Subramaniam K., Low W.Y., Lau P.C., Chin K.F., Chinna K., Kosai N., Taher M., Rajan R. (2018). Eating Behaviour Predicts Weight Loss Six Months after Bariatric Surgery: A Longitudinal Study. Nutrients.

[15] Mathisen T., Sundgot-Borgen J., Rosenvinge J., Bratland-Sanda S. (2018). Managing Risk of Non-Communicable Diseases in Women with Bulimia Nervosa or Binge Eating Disorders: A Randomized Trial with 12 Months Follow-Up. Nutrients.

[16] Nitsch M., Adamcik T., Kuso S., Zeiler M., Waldherr K. (2019). Usability and Engagement Evaluation of an Unguided Online Program for Promoting a Healthy Lifestyle and Reducing the Risk for Eating Disorders and Obesity in the School Setting. Nutrients.

[17] Burton A., Mitchison D., Hay P., Donnelly B., Thornton C., Russell J., Swinbourne J., Basten C., Goldstein M., Touyz S. (2018). Beliefs about binge eating: psychometric properties of the eating beliefs questionnaire (EBQ-18) in eating disorder, obese, and community samples. Nutrients.

[18] Amorim Palavras M., Hay P., Claudino A. (2018). An Investigation of the Clinical Utility of the Proposed ICD-11 and DSM-5 Diagnostic Schemes for Eating Disorders Characterized by Recurrent Binge Eating in People with a High BMI. Nutrients.

[19] ASTRA: A Centre for Translational Research and Action for Eating and Weight Disorders. https://www.westernsydney.edu.au/__data/assets/pdf_file/0007/1466377/Eating-Disorders_ICAS3065_White_Paper.pdf.

